# Sexual dysfunction in India

**DOI:** 10.4103/0019-5545.55948

**Published:** 2005

**Authors:** C. Andrade

**Affiliations:** Additional Professor

Sir,

Avasthi and Biswas^1^ presented a useful discussion on the current status of the pharmacotherapy of sexual dysfunction. In this context, I observe with surprise that there has been no survey on sexual dysfunction in India, much as there has been in other countries.^2^ Surveys of this nature are necessary to understand the magnitude and determinants of the problem, especially as may be regionally modulated by psychosocial, economic and possibly even biological factors.

To illustrate my meaning, I cite four hypotheses.

1. Indian men are at a higher risk of coronary heart disease than men in most other parts of the world; a biological risk factor has been postulated. The increase in risk is evident from the fourth decade of life onwards, when most men are still sexually active. As coronary heart disease and erectile dysfunction may share a common aetiology (atherosclerosis), erectile dysfunction attributed to atherosclerosis may be commoner in India than elsewhere in the world.
2. The average Indian woman exercises little and therefore, after an active life associated with studentship, tends to become sedentary. As a result, she tends to gain weight from the third decade of life onwards. This physical change is magnified after marriage by repeated childbirth. Furthermore, the average middle class Indian woman often dresses unattractively at home, such as in a billowing nightgown. These biological and behavioural factors could surely be expected to decrease the sexual appeal of a woman and increase the likelihood that her husband will experience erectile failure with her.
3. In urban India, women who work in software, business process outsourcing, and other organizations are empowered as never before. Their professional status and financial independence will encourage them to relate more aggressively with their spouses. Indian men are not used to leadership challenges from women in the family; the resultant relationship difficulties could increase the prevalence of erectile dysfunction.
4. In urban India, deadlines, targets, long working hours, night duties, and travel responsibilities in various professions ranging from information technology to pharmaceutical promotion could result in physical stress in the individual and emotional stress between a couple; the latter is especially likely when both spouses are employed. The result could be a variety of sexual dysfunctions, starting with a decreased libido.

Many other possibilies are conceivable, depending on the unique biological, socioeconomic, moral and behavioural subgroups in the melting pot of Indian cultures. Sexual function and dysfunction in India, with special reference to the long-neglected issue of female sexuality, require to be systematically investigated if this branch of medicine is to become relevant to the needs of the population.
